# Design and optimization of a shearing grinding device for efficient separation of eggshell and eggshell membrane

**DOI:** 10.3389/fbioe.2026.1928807

**Published:** 2026-09-21

**Authors:** Yuan Chi, Jiabao Lin, Yuxin Wang, Yuhao Jia, Yujie Chi

**Affiliations:** 1 College of Engineering, Northeast Agricultural University, Harbin, China; 2 College of Food Science, Northeast Agricultural University, Harbin, China

**Keywords:** biomass valorization, eggshell membrane, response surface optimization, separation rate, shearing grinding mechanism, single factor experiment

## Abstract

Global poultry egg production reached 100 million tonnes in 2024, and eggshell membranes account for approximately 10% of total egg mass. Mass egg consumption produces enormous amounts of waste eggshell. Eggshell is a natural calcium source, while eggshell membrane contains abundant proteins; both can be further processed into high value-added products. Current eggshell-membrane separation technologies commonly suffer from high energy input, chemical pollution or low separation efficiency, restricting large-scale industrial application. To address this bottleneck, this work adopts a combined shearing-grinding crushing strategy, which makes full use of the brittle nature of eggshell and high toughness of eggshell membrane. Key components of a custom shearing-grinding separation device were innovatively designed for this process. A four-factor three-level Box-Behnken response surface methodology (RSM) was implemented, with eggshell membrane separation rate selected as the response indicator for parameter optimization. The RSM-BBD regression model exhibited acceptable fitting performance (adjusted R^2^ = 0.8637, predicted R^2^ = 0.6983, Adequate Precision = 12.729). Under optimized operating conditions (15% moisture content, motor speed of 1,000 r/min, feed mass of 51 g, rotor-stator gap of 1 mm), a maximum separation rate of 87.28% was achieved. The proposed shearing-grinding device features simple structure, low energy consumption and environmental compatibility. This study offers a feasible technical route for the efficient industrial separation of waste eggshell and eggshell membrane, and supports the high-value valorization of poultry biomass waste within a circular economy framework.

## 1 Introduction

As an indispensable food in people’s daily diet, global poultry egg production reached 100 million tonnes in 2024 (Food and Agriculture Organization of the United Nations, 2024), with hen eggs accounting for 94% of the total output. Eggs are rich in nutrition and have a low cost. In recent years, demand for eggs as a raw material in the food industry has been steadily increasing (Yang et al., 2025). The discarded eggshell accounts for about 10% of the whole egg weight (Chi et al., 2022). Eggshell waste is often disposed of by direct landfilling, which may foster the growth of harmful microorganisms and pose potential risks to the surrounding ecological environment and human health (Vuong et al., 2018). Discarded eggshell and eggshell membrane are high-value biological wastes with broad application potential in multiple fields, and relevant research has confirmed their unique material properties and utilizable value. Eggshell, with a calcium carbonate content exceeding 94% (Choi et al., 2016), is a promising natural calcium source: in contrast to synthetic calcium supplements and oyster shell-derived calcium, eggshell-based calcium additives exhibit higher bioavailability and faster decomposition in artificial gastric juice, making them ideal for medicinal excipients and nutritional calcium supplements (Murakami et al., 2007; Siemiradzka et al., 2018; Haq et al., 2024; Dolińska et al., 2016). Beyond the food and pharmaceutical industries, eggshell powder can also improve the mechanical properties of building materials such as mortar and concrete, and its application in functional gypsum boards can mitigate sick building syndrome, realizing resource utilization in civil engineering (Omur et al., 2024; Tsubouchi et al., 2024). As a natural biopolymer material, eggshell membrane is primarily composed of keratin, chondroitin sulfate, and glycosaminoglycans (GAGs) including hyaluronan and keratan sulfate (Liu et al., 2014); these components endow it with diverse biological and industrial applications, such as skin care for improving skin elasticity and anti-aging effects (Yoo et al., 2014; Kalman and Hewlings, 2020), serving as a food-grade scaffold for cultured meat bovine muscle stem cell growth (Jang et al., 2024), acting as a biological dressing for burn and wound healing or a substrate for osteoblast proliferation (Aggarwal et al., 2024; Mensah et al., 2023), and even participating in environmental bioremediation for heavy metal removal (Mhd Wahi et al., 2025). Additionally, eggshell waste can be processed into alumina-supported carbon composites, and the optimization of eggshell and eggshell membrane ratios has been proven to significantly enhance fluorine adsorption performance of such materials (Lunge et al., 2012). Despite the abundant research on the resource utilization of eggshell and eggshell membrane, the practical application of these values is largely restricted by the lack of efficient, environment-friendly separation technologies for eggshell and eggshell membrane, which has become a key bottleneck limiting the large-scale utilization of this biological waste. Eggshell and eggshell membrane have extremely high application value, and the separation of eggshell and eggshell membrane is a prerequisite for effective utilization.

In recent years, various methods have been applied to separate eggshell and eggshell membrane, and there are three main approaches, namely: physical separation, chemical separation and biological separation. Yuan Chi et al. developed a mechanical stirring eggshell and eggshell membrane separation device, and found that the membrane recovery rate of the device was about 68% (Chi et al., 2019). However, the separation efficiency of this method was relatively low. They later designed a flash evaporation separation test device for eggshell membrane (Chi et al., 2023). By instantly reducing pressure, the superheated liquid inside the eggshell underwent flash evaporation, causing phase change and steam generation. The eggshell membrane was separated through the power of steam. However, heating may change the protein properties of the eggshell membrane. Using chemical separation of eggshell membrane can obtain a higher separation rate. However, this method will cause environmental pollution and the waste of eggshell resources. The chemical method entails immersing eggshells in acidic or alkaline solutions for separation (Homavand et al., 2024), yet this approach is prone to causing ecological environmental pollution, altering the physicochemical properties of eggshell membranes, and thus impeding the subsequent application of eggshell membranes. Moreover, the prolonged chemical reaction time leads to an extended shell-membrane separation cycle. The enzymatic method, by contrast, achieves eggshell membrane separation through protease-mediated hydrolysis (Neunzehn et al., 2015). This method, however, requires rigorous reaction conditions for effective implementation, and the high cost of proteases also adds to the overall production expenses of eggshell membrane separation. To provide a clear and quantitative overview of the performance characteristics of the aforementioned separation methods, their key technical and economic indicators are summarized and compared in Table 1.

**TABLE 1:** Comparison of key performance indicators for eggshell and eggshell membrane separation methods.

Separation method	Typical separation rate	Operation cost	Environmental impact
Physical (mechanical stirring)	68%	Low	Negligible
Physical (flash evaporation)	73.48%	Moderate	Negligible
Chemical (acid/alkaline immersion)	90%	Moderate	Severe pollution (acid/alkaline wastewater discharge); eggshell resource waste
Biological (enzymatic hydrolysis)	88%	High	Negligible

Through preliminary experiments, it was found that broken eggshell and eggshell membrane had a better separation effect. The working principle is to utilize the brittleness of eggshell and the toughness of eggshell membrane to separate them. However, most crushers are mainly designed to process bulk minerals, special waste, grains, and medicinal herbs. There is no specialized equipment for crushing eggshell and eggshell membrane (Han et al., 2023; Rivera-García et al., 2023; Liu et al., 2024).

This study aimed to develop a laboratory-scale device integrating shear and grinding mechanisms, quantify the effects of moisture content, rotor speed, feed mass, and rotor-stator gap, and validate an RSM-derived operating condition through independent confirmation experiments.

Most existing eggshell separation devices adopt single impact crushing or simple screening structures, which cannot fully utilize the mechanical property differences between brittle eggshell and tough eggshell membrane, often resulting in high energy consumption and incomplete separation. Differing from the reported equipment, the proposed customized shearing-grinding device couples shear and grinding effects through optimally designed rotor and stator components. This structural design matches the intrinsic mechanical characteristics of eggshell and eggshell membrane, realizing physical separation without chemical additives. The main scientific contribution of this work lies in providing a dedicated mechanical separation strategy and optimized structural parameters for eggshell resource valorization, which supports clean and low-cost eggshell membrane separation.

## 2 Materials and methods

### 2.1 Egg samples

In this study, 30 fresh Hy-Line Brown eggs were randomly selected for the measurement of physical parameters. Egg yolk and albumen were removed, and eggshells were thoroughly washed with tap water to eliminate residual albumen and surface contaminants. External dimensions were measured using a vernier caliper (accuracy: 0.02 mm). The average long axis length was 58.04 mm, short axis length was 45.30 mm, mean eggshell thickness was 0.34 mm, and mean eggshell membrane thickness was 0.10 mm. All measurements were performed in triplicate to ensure reliability.

Eggs were freshly purchased from a local large-scale supermarket. According to the supplier, eggs were procured on the purchase day and used promptly under room temperature. Thirty eggs were only for dimensional measurement. Sufficient eggs were prepared for all experiments, and samples were randomly selected. All experiments were performed in three independent replicate groups.

### 2.2 Shearing grinding experiment equipment

The separation of eggshell and eggshell membrane was investigated using a lab-scale shearing grinding device. Figure 1 presents a schematic diagram of the experimental equipment, which comprises three main components: the control section (frequency converter), power section (motor), and working section (crusher). Additional equipment included a three-phase asynchronous motor (Y80M2-4), frequency converter (Delta VFD-M), cyclone separator for sample collection, and electronic balance (FA2004B). The crusher structure is illustrated in Figure 2.

**FIGURE 1:**
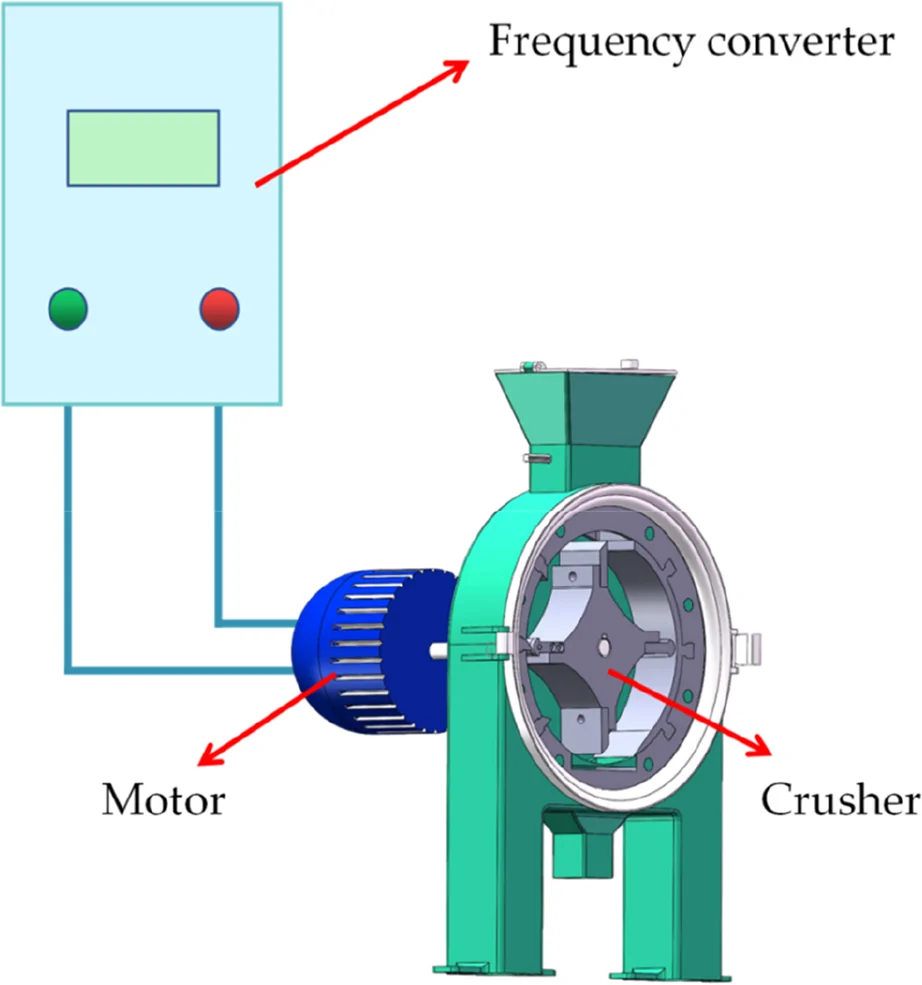
The schematic diagram of shearing-grinding experiment equipment.

**FIGURE 2:**
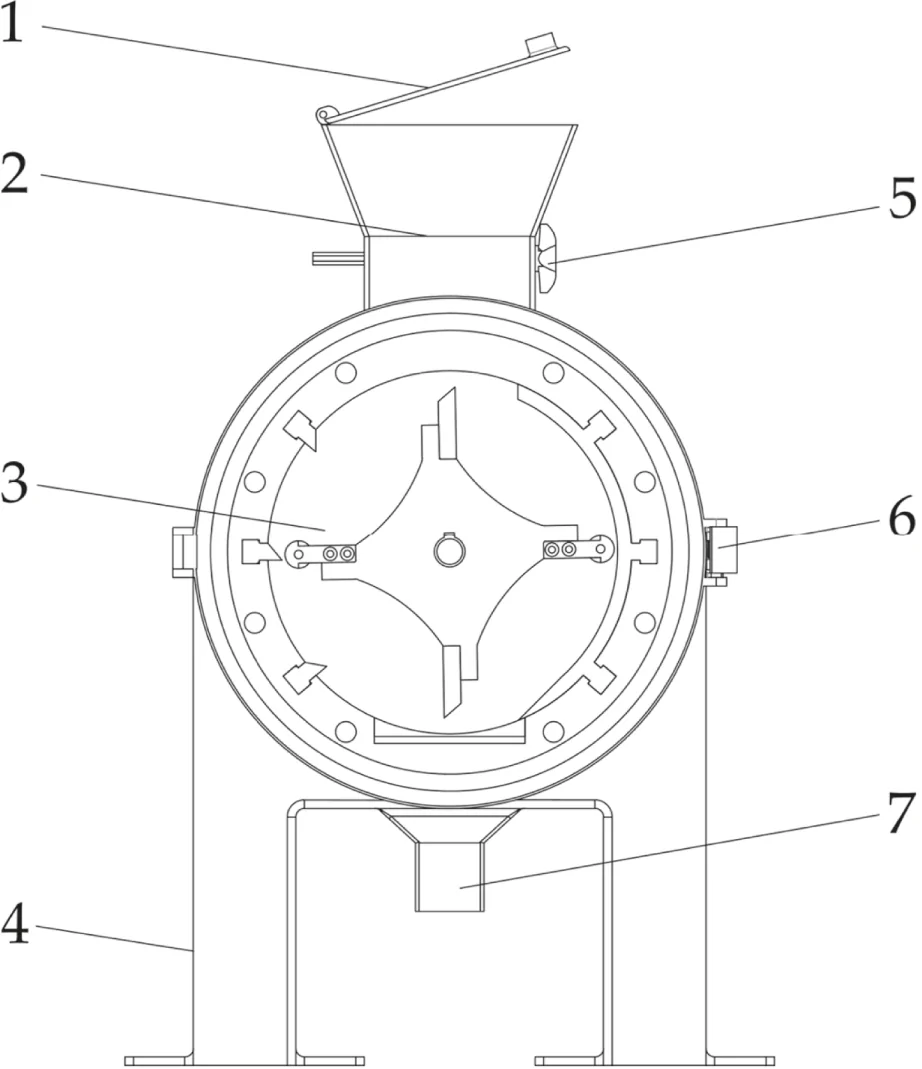
The structure of the crusher. 1. Hopper cover 2. Hopper 3. Crushing chamber 4. Machine frame. 5. Rotary knob 6. Locking clamp 7. Discharge hole.

The shearing blade is a key component affecting the separation efficiency of eggshell and eggshell membrane. Its structural parameters directly determine the shearing force and separation effect. Referring to the design experience of agricultural and food processing equipment (Wu et al., 2025), combined with the mechanical characteristics of eggshell and eggshell membrane, the shearing blade is designed as a rectangular structure with a length of 42 mm, a width of 50 mm, and a thickness of 10 mm. The blade edge is polished to ensure smooth shearing without damaging the eggshell membrane. The material of the shearing blade is selected as 304 stainless steel, which has good corrosion resistance and wear resistance, and can avoid rust pollution during long-term use.

The key components of the crushing and separation device are illustrated in Figure 3. The apparatus comprises seven main parts: (1) hopper cover, (2) hopper, (3) crushing chamber, (4) machine frame, (5) rotary knob, (6) locking clamp, and (7) discharge hole. The stator was manufactured via 3D printing, whereas remaining components were machined from 304 stainless steel. The stator contains six grooves to accommodate three static shearing blades and a rolling ring. The rotor is equipped with two rotational shearing blades and two grinding blades, all bolted to the rotor. Grinding blades and rolling ring collectively facilitate the rolling action of eggshell and eggshell membrane.

**FIGURE 3:**
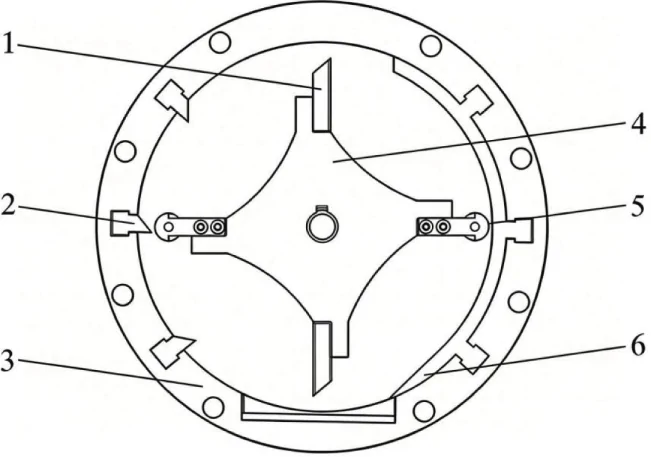
Structure drawing of key components of the device for separating eggshell and eggshell membrane (1). 1. Rotational shearing blade 2. Static shearing blade 3. Stator 4. Rotor 5. Grinding blade 6. Rolling ring.

#### 2.2.1 Design of the shearing blades

Tool design requires consideration of three primary factors: cutting force, abrasion resistance, and chip evacuation. The material selected for this study was discarded eggshell, characterized by high eggshell brittleness and high membrane toughness. Smaller edge angles generate higher cutting forces, making them suitable for tough materials, but exhibit lower abrasion resistance. Conversely, larger edge angles offer superior abrasion resistance and are appropriate for brittle materials, but produce lower cutting forces. Preliminary experiments were conducted at 20 °C to evaluate the effects of 50° and 70° shearing blades on eggshells. Blade thickness was 10 mm. When the temperature was 20 °C and the gap between rotor and stator was 2 mm, the 50° shearing blade was more effective than 70° shearing blade in separating eggshell and eggshell membrane. The main reason was that the 50° shearing angle was relatively small and the corresponding shearing plane area was larger for blades with identical thickness. The small shearing angle prolonged the effective shearing process, consequently improving material fragmentation efficiency. At the same time, it also increased the range of the shearing action and destroyed the adhesive force between eggshell and eggshell membrane more effectively. Meanwhile, it was also more conducive to the grinding separation of eggshell and eggshell membrane. Therefore, taking the above reasons into account, the selected shearing blade angle was 50°.

#### 2.2.2 Design of the grinding blade

The end roller of the grinding blades exhibited two movement modes: rolling and sliding (Figure 4). These modes generated distinct forces on discarded eggshells between the grinding blades and rolling ring. In rolling mode, blade rotation produced minimal friction between the end roller and eggshell, exerting negligible influence on eggshell movement. Conversely, sliding mode generated substantially greater friction, significantly affecting eggshell movement dynamics.

**FIGURE 4:**
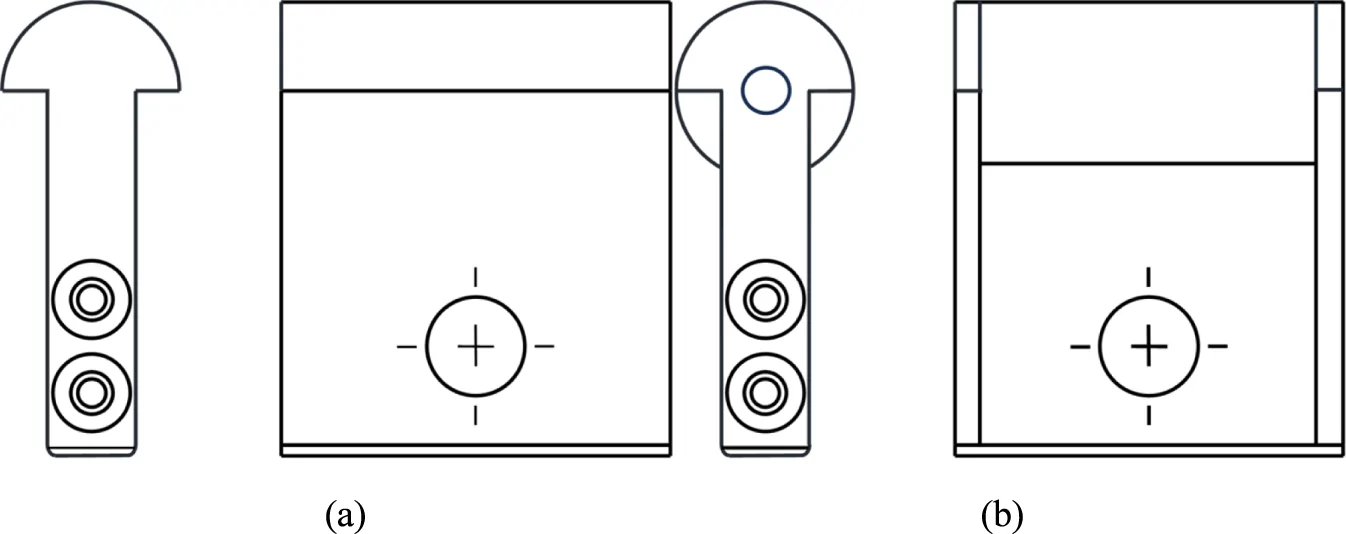
The movement form of grinding blades. **(a)** Sliding form; **(b)** Rolling form.

In this paper, the rotor rotated clockwise. When the end roller was designed in rolling form. Figure 5a shows the schematic diagram of the forces on discarded eggshell between the grinding blade and the rolling ring, which contained the gravity on the discarded eggshell (*F*_*G*_), the pressure of the grinding blade on the discarded eggshell (*F*_*A*_), the friction between the rolling ring and the discarded eggshell (*F*_*f*_), and the pressure of the rolling ring on the discarded eggshell (*F*_*n*_). The friction between the grinding blade and the eggshell was rolling friction, which had little effect on the eggshell and was therefore ignored in this paper. The angle between *F*_*A*_ and the vertical direction was *δ*. Between the grinding blade and the rolling ring, the discarded eggshell was crushed instantly under the action of the two components and the time was very short. At this stage, the crushed eggshell remained attaching to the rotor, thus maintaining their original rotational motion, the crushing process can be approximated as uniform circular motion, The centripetal force (*F*) was provided by the combined force of *F*_*n*_ and *F*_*A*_.

**FIGURE 5:**
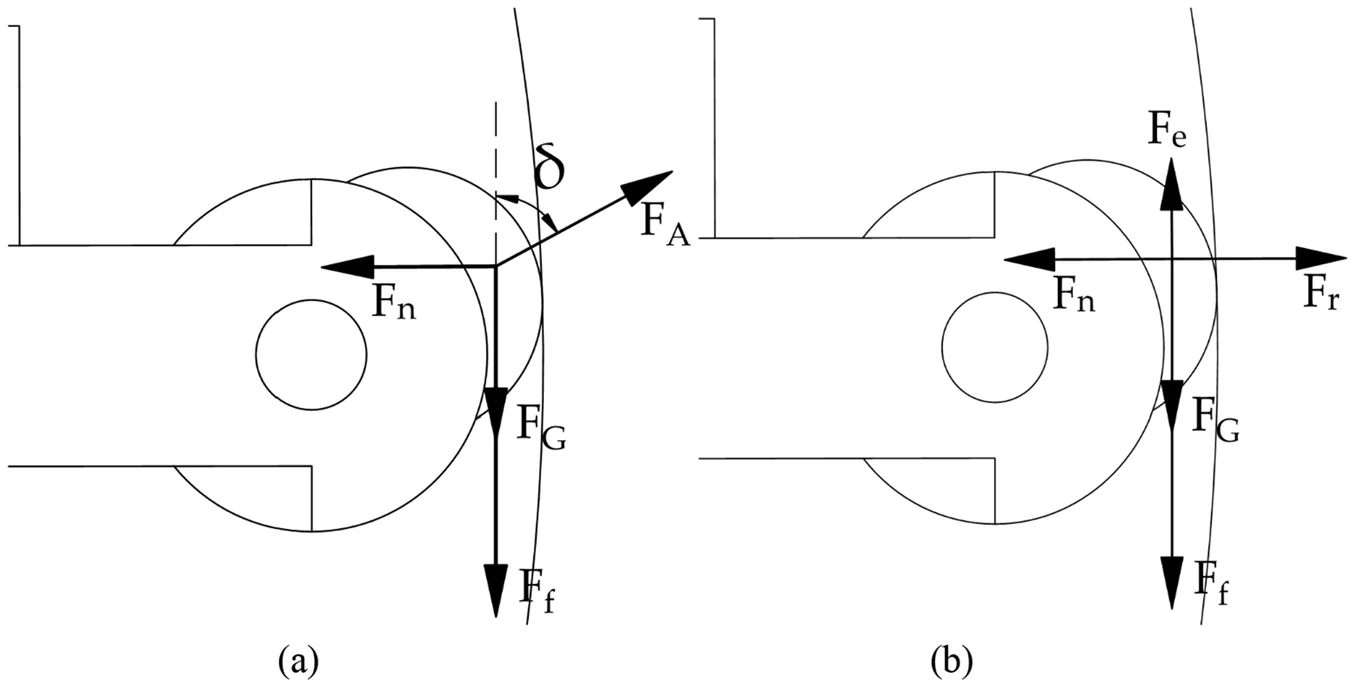
Analysis of eggshell stress under rolling conditions. **(a)** Before force decomposition. **(b)** After force decomposition.

The acting force *F*_*A*_ on the discarded eggshell was mainly related to the motor rotational speed *n* and the motor output power *P*. The relationship between motor output power and torque was given in Equation 1, as stated below (Krogstad and Lund, 2012).

$$T=\frac{9550P}{n}$$ (1)

where *P* is the output power (kW); *T* is the motor torque (N⋅M); *n* is the motor rotational speed (r/min).

The discarded eggshell particles are impacted by the rotor as they move within the crusher, and in this process, the speed of eggshell particles varies due to air resistance, friction and collision. The rotor was connected with the motor, and the speed remained unchanged. *v*_*n*_ is defined as the tangential velocity component at the blade tip. The *v*_*n*_ was calculated using Equation 2, as stated below (Li et al., 2024).

$$v_{n}=2\pi nr$$ (2)

where *r* is the radial distance from the blade tip to the spindle axis.

When crushing, the discarded eggshell was mainly affected by the pressure of the grinding blades and the friction of the rolling ring. They did not slide relative to the grinding blades and the rolling ring. The gravity could be neglected. The *F*_*A*_ was calculated using Equation 3, as stated below.

$$F_{A}=\frac{T}{Nr}=\frac{9550P}{nNr}$$ (3)

where *N* denotes the instantaneous hypothetical number of eggshell fragments under extrusion at a certain moment inside the crushing chamber. This parameter is introduced only for establishing the mechanical force balance model and cannot be experimentally quantified with our current setup. It does not participate in the calculation of the core experimental index.

This equation assumes torque is evenly distributed among all N fragments to simplify theoretical derivation. In practice, torque and extrusion force acting on individual eggshell fragments are unevenly distributed due to random collisions, overlapping extrusion and irregular fragment geometry.

The *F*_*A*_ was decomposed along the horizontal and vertical directions as shown in Figure 5b. The angle between *F*_*A*_ and the vertical direction was *δ*, the horizontal force decomposed was Fr and the vertical force decomposed was *F*_*e*_. The combined force of *F*_*r*_ and *F*_*n*_ provided the centripetal force (*F*). The centripetal force was calculated using Equation 4, The component forces were shown in Equation 5, as stated below.

$$F_{A}=F_{n}-F_{r}=m\frac{v_{n}^{2}}{r}$$ (4)

where m is the mass of a single eggshell fragment undergoing uniform circular motion within the grinding gap (kg).

$$\left\{\begin{array}{c} F_{e}=F_{A}\cos\delta \\ F_{r}=F_{A}\sin\delta \end{array}\right.$$ (5)

When the end roller was designed in sliding form, there was friction between the grinding blades and the discarded eggshell. The diagram of force of the discarded eggshell under the action of the grinding blade is shown in Figure 6a. The forces on the eggshell included: the gravity on the discarded eggshell (*F*_*G*_), the pressure of the grinding blade on the discarded eggshell (*F*_*A*_), the friction between the rolling ring and the discarded eggshell (*F*_f1_), the pressure of the rolling ring on the discarded eggshell (*F*_*n*_) and the friction between the grinding blade and the discarded eggshell (*F*_f2_). The angle between *F*_*A*_ and the vertical direction was *θ*. The angle between *F*_f2_ and the vertical direction was *α*. The centripetal force (*F*) was provided by the combined force of *F*_*n*_, *F*_f2_ and *F*_*A*_.

**FIGURE 6:**
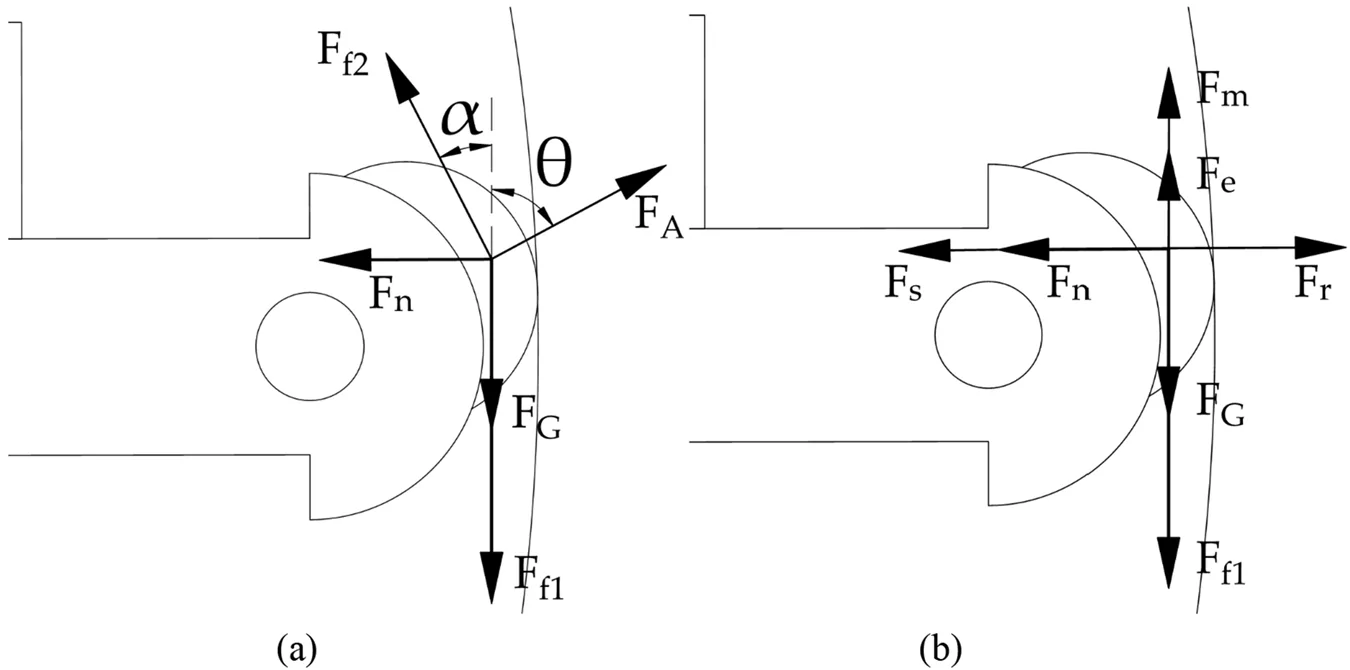
Analysis of eggshell stress under sliding conditions. **(a)** Before force decomposition. **(b)** After force decomposition.

When crushing, the discarded eggshell was mainly affected by the pressure of the grinding blade and the friction of the rolling ring, and gravitational effects were negligible. When the eggshell entered the gap between the grinding blade and the rolling ring, due to the extrusion of both components and the sliding friction, the friction increased. The grinding blade can drive the eggshell for uniform circular motion, the formula of *F*_*A*_ was calculated using Equation 6, as stated below.

$$F_{A}=\frac{T}{Nr}=\frac{9550P}{nNr}$$ (6)

The *F*_*A*_ was decomposed along the horizontal and vertical directions as shown in Figure 6b The horizontal force decomposed was *F*_*r*_ and the vertical force decomposed was *F*_*e*_. The *F*_f2_ was also decomposed along the horizontal and vertical directions. The horizontal force decomposed was *F*_*s*_ and the vertical force decomposed was *F*_*m*_. The combined force of *F*_*r*_, *F*_*n*_ and *F*_*s*_ provided the centripetal force (*F*). The centripetal force was calculated using Equation 7, as stated below. The component forces were shown in Equation 8, as stated below.

$$F_{A}=F_{n}+F_{s}-F_{r}=m\frac{v_{n}^{2}}{r}$$ (7)

$$\left\{\begin{array}{c} F_{e}=F_{A}\cos\theta \\ F_{r}=F_{A}\sin\theta \\ F_{s}=F_{f2}\cos ɑ\\ F_{m}=F_{f2}\sin ɑ \end{array}\right.$$ (8)

It can be seen from Equation 6 that the crushing force on discarded eggshell within the crusher was mainly determined by the output power of the motor. The output power of the motor was proportional to the torque. When other conditions remain unchanged, the greater the power of the motor, the greater the impact of the grinding blade on the eggshell. When the output power was low, the impact force was not enough and the eggshell may not be crushed sufficiently; conversely, when the output power was high, the impact force was large. Excessive crushing of eggshell will produce a large amount of fine eggshell powder, which is easy to cause air dust pollution during the separation process and increase the difficulty of subsequent solid-liquid separation if water is involved, leading to secondary environmental pollution. Therefore, the appropriate power range should be selected according to the test, so as to ensure the normal operation of subsequent sorting.

In order to obtain a roller radius with a higher separation rate, pre-experiments were conducted using kraft paper circular tubes of different radii (6, 8, 10, 12 mm), as shown in Figure 7. Firstly, fresh eggshells were washed and dried, then divided into four groups (*n* = 5 replicates per group). Secondly, each group was ground using cylindrical barrels of a specific radius. The other portion of eggshells was initially weighed and reweighed after full drying to calculate the water content following the eggshell membrane water measurement method detailed in Section 2.4. Finally, the eggshell membrane separation rate was determined for each group to quantitatively evaluate the grinding effect. The quantitative results of separation rates for each roller radius group are presented in Table 2.

**FIGURE 7:**
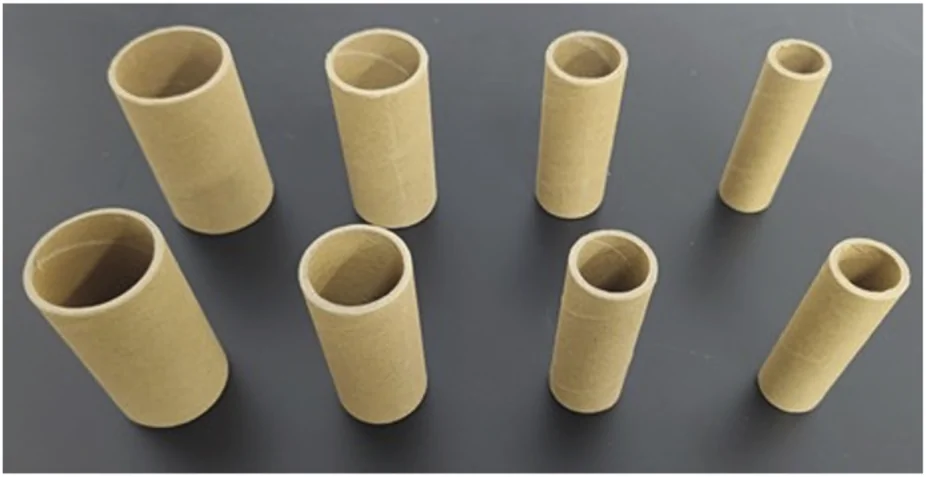
Kraft paper circular tubes with different radii.

**TABLE 2:** Separation rates of eggshell membrane under different roller radii.

Roller radius (mm)	Separation rate (mean ± SD, %)
6	68.4 ± 3.2
8	82.7 ± 2.5
10	75.3 ± 2.8
12	70.1 ± 3.0

Statistical analysis was performed using SPSS 26.0 software. One-way analysis of variance (One-way ANOVA) followed by Tukey HSD *post hoc* test was used to compare the separation rates among different roller radii. The results showed that the roller radius had a significant effect on the separation rate (*F* = 18.64, df = 3, 16, *P* < 0.0001). The separation rate of the 8 mm radius group (82.7% ± 2.5%) was significantly higher than that of the 6 mm (68.4% ± 3.2%, *P* < 0.001), 10 mm (75.3% ± 2.8%, *P* = 0.012), and 12 mm (70.1% ± 3.0%, *P* = 0.003) groups. Therefore, a roller radius of 8 mm was selected for the grinding blade in this study. The overall dimensions of the grinding blades were the same as those of the shearing blades to prevent teeth collision, namely, a length of 42 mm, a width of 50 mm, and a thickness of 10 mm, ensuring the normal operation of the crusher.

### 2.3 Design of the rolling ring

Traditional crushers use shearing methods. This study utilized the high brittleness of eggshell and the high toughness of eggshell membrane, adopting a combined shearing and grinding crushing and separation method. Pure shearing methods might cause significant damage to egg membrane, making posterior collection difficult. It might also result in eggshell fragment still adhering to under-removed eggshell membrane, leading to lower separation rate. Therefore, adopting a combined shearing and grinding method could enhance separation rate and facilitate subsequent operations.

Due to the integration of grinding blades into the device, an excessive gap existed between the stator region without stationary shearing blades, as illustrated on the right side of Figure 3, and the rotating grinding blades. This gap would result in incomplete crushing of discarded eggshells because partial materials might pass through without sufficient shearing and grinding actions. To address this issue, a rolling ring was specially designed and installed to compensate for the installation distance and ensure a uniform working gap in the crushing chamber, as shown in Figure 8. The rolling ring is fabricated with 304 stainless steel and is consistent with the main structural materials of the device. Its detailed dimensional parameters are as follows: an outer diameter of 320 mm, an inner diameter of 280 mm and a thickness of 50 mm. The protruding height of the rolling ring is 10 mm and is completely consistent with that of the stationary shearing blades. This design guarantees a consistent and appropriate working gap between the rotor and stator throughout the entire crushing chamber. It effectively eliminates the uncrushed dead zone and ensures thorough crushing of discarded eggshells.

**FIGURE 8:**
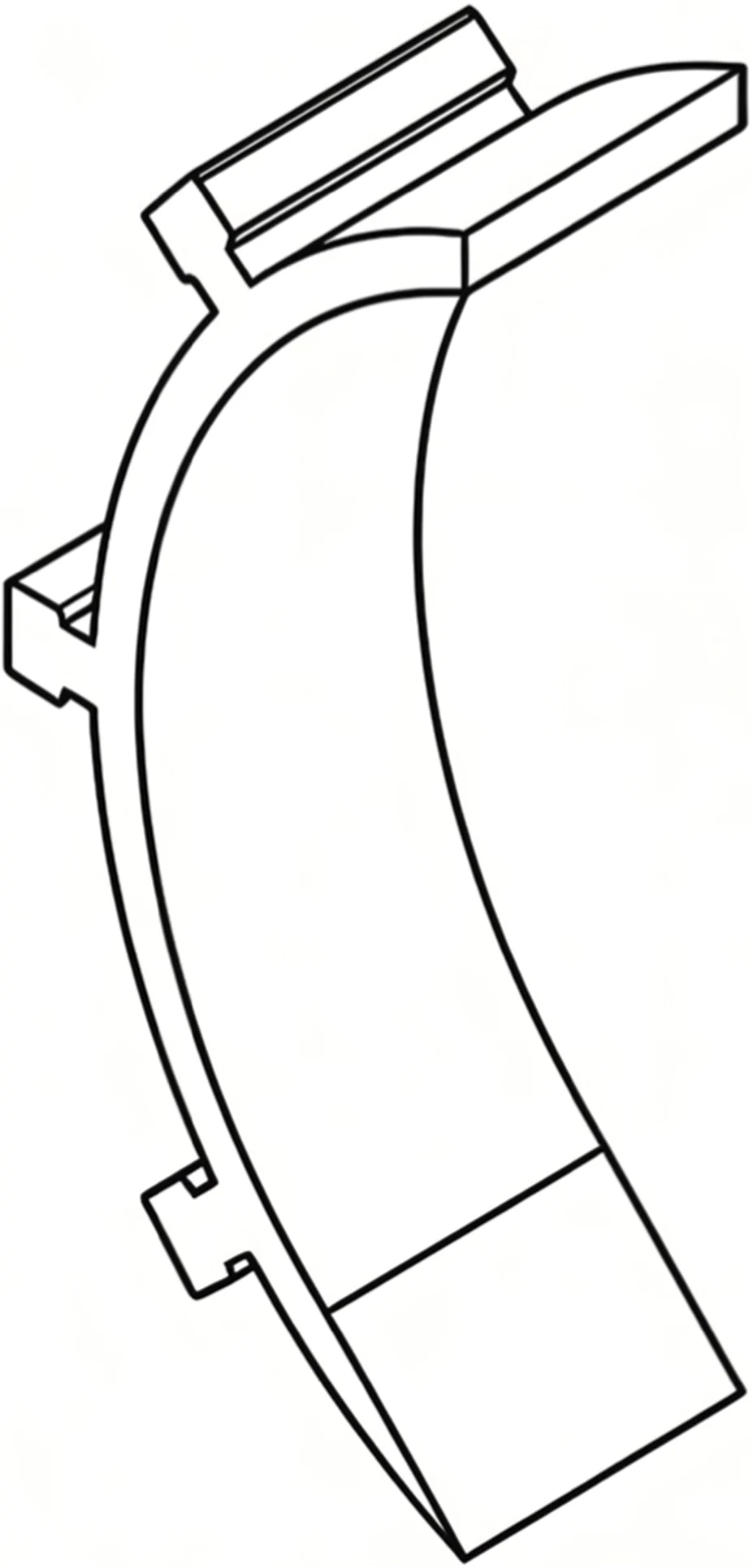
The shape of rolling ring.

### 2.4 Experimental method

As shown in Figure 1, the shearing grinding experiment equipment was designed to investigate the effect of the key factors on the separation rate. The experimental process mainly included three steps: namely feeding, crushing and unloading. First, under the condition of room temperature, a specified mass of cleaned and discarded eggshell was fed into the device through the hopper. Seal the whole equipment by rotary knob to prevent material from flying out during the working process. Then, the motor and frequency converter were started to bring the rotor up to the specified speed. The eggshell was separated through the function of shearing and grinding. When the crushing process was complete, open the discharge hole of the device. Finally, the separated eggshell and eggshell membrane were collected and left to dry naturally at a room temperature of 20 °C. All membrane samples were treated under identical ambient conditions with the same air-drying workflow prior to weighing to guarantee comparability among experimental groups. The eggshell membrane was sorted out through the collecting device and weighed using the electronic balance to calculate the separation rate. The experimental device is shown in Figure 9.

**FIGURE 9:**
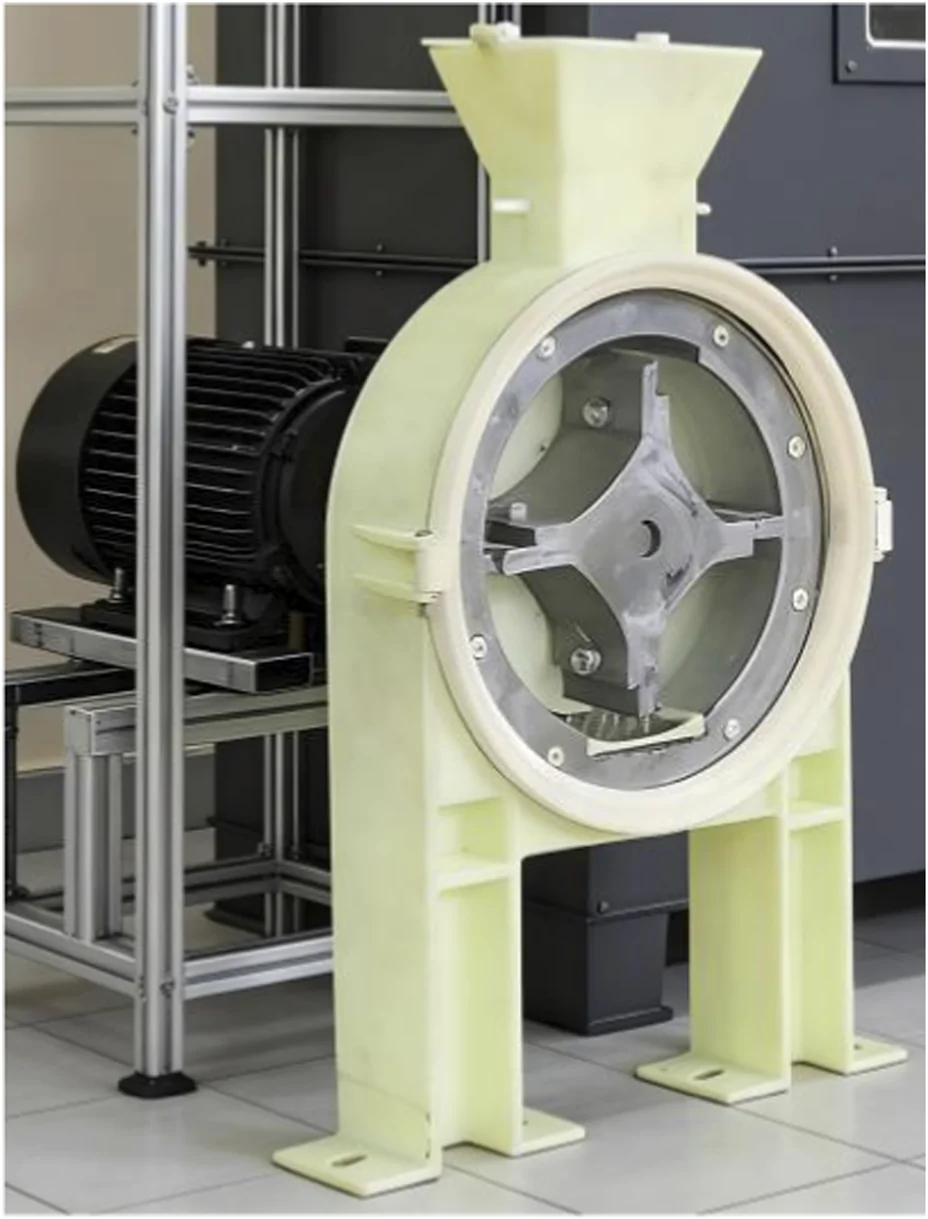
The experimental device.

In order to prevent dust pollution during the crushing of the discarded eggshell, the feeding hopper of the device adopted a closed design. After measurement, the separation rate was taken as the experimental index. By reviewing the relevant literature, the separation rate of eggshell and eggshell membrane can be expressed using the following formula (Chi et al., 2023), in which the constant term 0.03043 was calibrated through systematic eggshell separation experiments. The detailed expression is shown in Equation 9 (Chi et al., 2023):

$$Y=\frac{m}{0.03043M}\times 100\%$$ (9)

where *Y* is the separation rate (%); *M* is the mass of discarded eggshell (g); *m* is the mass of egg membrane after experiment (g).

All experiments were repeated three times, and the average value was taken as the final result. The random error was controlled by using the same experimental equipment and operating procedures, and the systematic error was corrected by calibrating the electronic balance, frequency converter and other instruments before the experiment.

### 2.5 Experimental design

#### 2.5.1 Single-factor experiment

In this paper, all experimental results were evaluated using the separation rate as the experimental index. The shearing grinding experimental factors included water content (0, 5, 10, 15%), motor rotational speed (600, 750, 900, 1,050 r/min), feeding amount (45, 50, 55, 60 g), gap between rotor and stator (1, 2, 3, 4 mm). The effect of different experimental factors on the separation rate was investigated utilizing the shearing grinding experiment equipment.

#### 2.5.2 Orthogonal experiment optimization

The single-factor experiments can identify which experimental factors have a significant effect on the separation rate. The optimal ranges of each factor obtained from single-factor experiments were then used for response surface methodology (RSM) based on Box-Behnken design (BBD). The Box-Behnken design was established using Design-Expert 13 software with the separation rate as the response index. This approach was adopted to analyze the interactive effects among experimental factors on the separation rate and to determine the optimal factor level combination for maximizing the separation rate in the shearing grinding process.

## 3 Results

### 3.1 Single-factor experimental results and analysis

#### 3.1.1 Effect of water content

Higher moisture content may increase the toughness of the eggshell membrane, thereby affecting its separation from the eggshell during processing. The moisture content of the cleaned discarded eggshell was determined following the national standard GB/T 6971—2007, *Test Methods for Feed Grinders* as expressed in Equation 10:

$$W=\frac{G-G_{0}}{G}$$ (10)

where *W* is the water content of discarded eggshell (%); *G* is the wet weight of the sample (g); *G*_0_ is the dry weight of the sample (g).

By reviewing the relevant research literature on eggshell membrane separation, the process time of shearing and grinding was set to 17 min in this study (Lunge et al., 2012). The water content of the fresh cleaned eggshell membrane was calculated to be about 13%. Therefore, the range of water content was selected as 0%–15%.

The raw eggshell exhibited an initial moisture content of approximately 13%. Samples with target moisture contents of 0%, 5%, 10%, and 15% were prepared gravimetrically with tap water. The 0% sample was obtained by drying raw eggshells. Tap water was quantitatively added to dried eggshells to produce 5% and 10% moisture samples, while the 15% moisture sample was prepared by supplementing extra tap water into raw eggshells. Each sample was fully mixed and equilibrated before experiments.

Under the conditions of a temperature of 20 °C, a motor rotational speed of 750 r/min, a feeding amount of 60 g, and a gap between rotor and stator of 2 mm. The effect of water content on the separation rate was studied at 0, 5, 10, 15%, respectively. Statistical analysis was performed using one-way ANOVA and Duncan’s test (*P* < 0.05). Data are expressed as mean ± SD (*n* = 3). The results are shown in Figure 10a. The separation rate was highest when the water content was 15%. There was significant increase in the separation rate at 15% water content compared to 0%. When the water content was relatively low, the brittleness of the eggshell membrane was high, and it was easy to break into tiny fragments during the crushing process, making it difficult to separate effectively from the eggshell (Bekeshova et al., 2022). When the water content became high, the appropriate water could play the role of lubrication and moistening. The eggshell membrane was not easy to break into tiny pieces during the crushing process. Therefore, the separation would proceed more thoroughly with increasing water content. Considering the operability of the shearing grinding experiment equipment, the optimal water content range was selected as 5%–15%.

**FIGURE 10:**
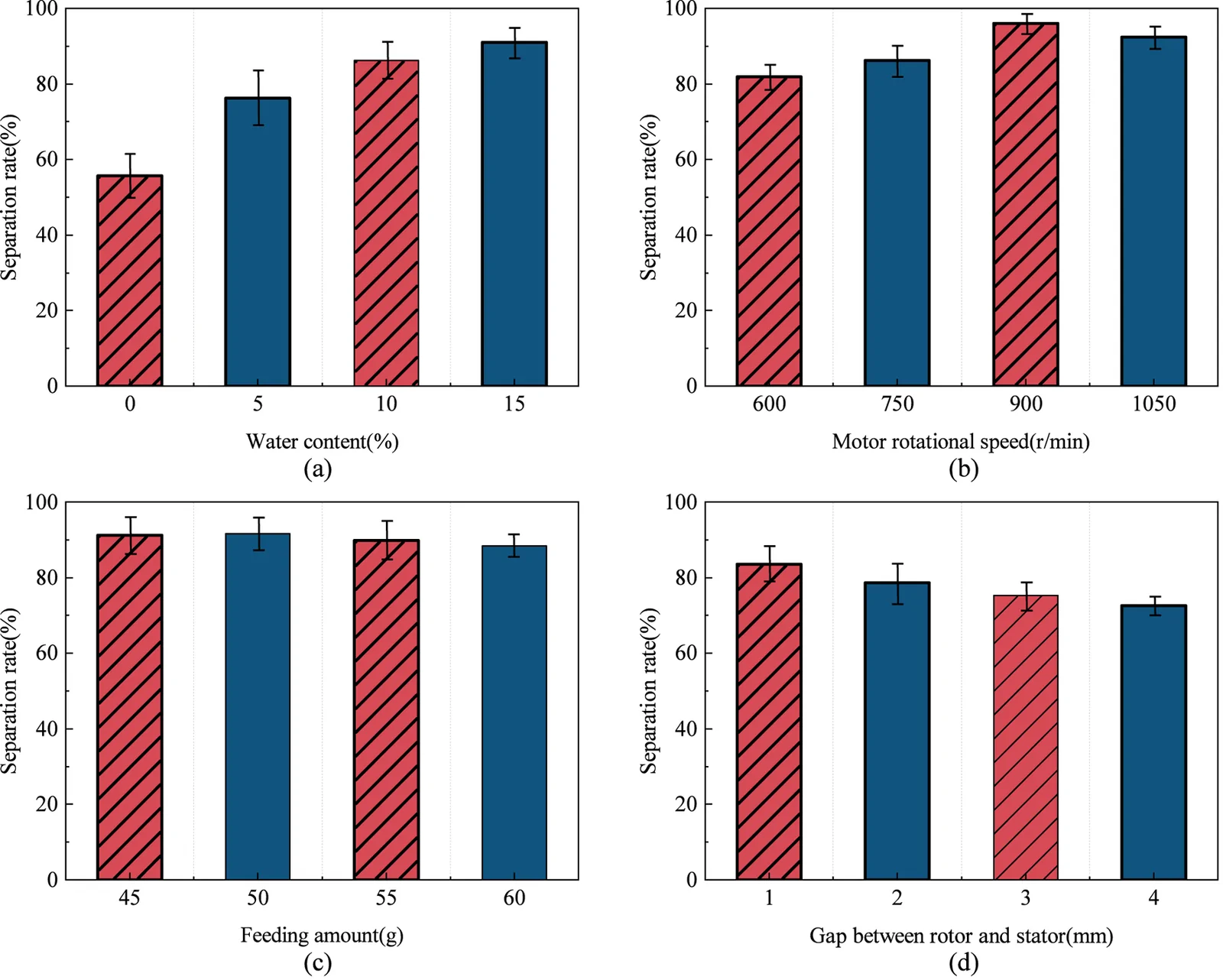
Effect of water content, motor rotational speed, feeding amount, and gap between rotor and stator on separation rate. **(a)** Water content. **(b)** Motor rotational speed. **(c)** Feeding amount. **(d)** Gap between rotor and stator.

#### 3.1.2 Effect of motor rotational speed

Statistical analysis was performed using one-way ANOVA and Duncan’s test (*P* < 0.05). Data are expressed as mean ± SD (*n* = 3). Figure 10b shows the effect of motor rotational speed at 600, 750, 900, 1,050 r/min on the separation rate under the conditions of a temperature of 20 °C, a water content of 10%, a feeding amount of 60 g, and a gap between rotor and stator of 2 mm. As the motor rotational speed increased, the impact of the rotor on the discarded eggshell was much greater, and eggshell was subjected to more intense shearing and grinding effects. The separation rate showed considerable variation as the motor rotational speed changed from 600 r/min to 1,050 r/min, as illustrated in Figure 10b. The increase of speed had a positive effect on the shearing grinding experiment process. However, excessive speed may generate additional heat, which could potentially lead to protein denaturation and reduced water content of the discarded eggshell. Also, high speed may result in too much powder (Chen et al., 2023). Therefore, taking into account the above reasons and the laboratory operating environment, the optimal experimental speed range was selected as 750 r/min −1,050 r/min.

#### 3.1.3 Effect of feeding amount

Statistical analysis was performed using one-way ANOVA and Duncan’s test (*P* < 0.05). Data are expressed as mean ± SD (*n* = 3). The effects of feeding amount of 45, 50, 55 and 60 g on the separation rate at 20 °C, 10% water content, 750 r/min motor rotational speed, and a gap between rotor and stator of 2 mm are shown in Figure 10c. Statistical analysis was performed using one-way ANOVA followed by Duncan’s multiple comparison test (*P* < 0.05), and data are presented as mean ± SD of three independent replicates. The feeding amount was negatively correlated with the separation rate, and there were significant differences in separation rate among different feeding amount groups. Excessive feeding amount made it easier for discarded eggshell to clog between the rotor and stator, reducing the possibility of contact between eggshell and blades, resulting in insufficient crushing of the eggshell and decreasing the separation rate. In addition, since the output power of the motor was certain, when the mass of eggshell increased, the energy gain per unit mass was reduced, so the separation rate was decreased. Consequently, this paper set the optimal feeding amount range at 45–55 g to ensure the operability and separation effect of this experiment.

#### 3.1.4 Effect of the gap between rotor and stator

The effects of gap between rotor and stator of 1, 2, 3 and 4 mm on the separation rate at 20 °C, 10% water content, 60 g feeding amount and 750 r/min motor rotational speed are illustrated in Figure 10d. Statistical analysis was performed using one-way ANOVA and Duncan’s test (*P* < 0.05). Data are expressed as mean ± SD (*n* = 3). When the gap was larger, some of the waste eggshell passed directly through the gap and cannot undergo effective shearing and grinding action, so the separation rate decreased gradually as the gap increased. The size of gap between the rotor blades and the fixed blades will directly affect the quality of the crushed material. Considering the above reasons, the range of gap between rotor and stator was selected as 1–3 mm in this study.

### 3.2 Response surface optimization

To further investigate the effects of the parameter levels of this experiment on eggshell membrane separation, the optimal parameter combination and interaction of the influencing factors on the experiment index were investigated. Water content *X*_1_, motor rotational speed *X*_2_, feeding amount *X*_3_ and gap between rotor and stator *X*_4_ were used as experimental factors. The separation rate Y was used as experimental index. A four-factor and three-level combination experimental design was adopted according to the Box-Behnken design (BBD) test principle. Combining the results of single factor experiment and the existing conditions in the laboratory, the water content of 5%–15%, motor rotational speed of 750–1,050 r/min, feeding amount of 45–55 g, gap between rotor and stator of 1–3 mm were selected. The BBD levels were three (−1, 0, +1) for each significant factor. The level coding for each factor is shown in Table 3. Table 4 illustrates the experimental design and results.

**TABLE 3:** Levels of the experimental factors.

Level	Water content *X*_1_ (%)	Motor rotational speed *X*_2_ (r/min)	Feeding amount *X*_3_ (g)	Gap between rotor and stator *X*_4_ (mm)
−1	5	750	45	1
0	10	900	50	2
1	15	1,050	55	3

**TABLE 4:** Experimental results of response surface.

Run	*X*_1_ (%)	*X*_2_ (r/min)	*X*_3_ (g)	*X*_4_ (mm)	*Y* (%)
1	5	750	50	2	72.38
2	15	750	50	2	74.58
3	5	1,050	50	2	75.46
4	15	1,050	50	2	85.22
5	10	900	45	1	75.90
6	10	900	55	1	80.83
7	10	900	45	3	71.92
8	10	900	55	3	77.69
9	5	900	50	1	80.26
10	15	900	50	1	88.62
11	5	900	50	3	76.12
12	15	900	50	3	77.68
13	10	750	45	2	72.31
14	10	1,050	45	2	74.06
15	10	750	55	2	73.66
16	10	1,050	55	2	82.23
17	5	900	45	2	71.69
18	15	900	45	2	80.05
19	5	900	55	2	74.32
20	15	900	55	2	83.21
21	10	750	50	1	79.33
22	10	1,050	50	1	83.95
23	10	750	50	3	73.02
24	10	1,050	50	3	77.53
25	10	900	50	2	80.84
26	10	900	50	2	82.16
27	10	900	50	2	84.27
28	10	900	50	2	83.65
29	10	900	50	2	85.68

The regression analysis of the experimental results was conducted using Design-Expert 13 software, and a quadratic polynomial regression equation for the eggshell membrane separation rate Y was fitted. The analysis of variance (ANOVA) and significance tests were performed for the regression equation, with the results shown in Table 5. As presented in Table 5, the model had a P-value <0.01, indicating a highly significant regression model, and the lack of fit term was insignificant (*P* = 0.6169 > 0.05), suggesting a good fitting effect of the regression equation. The adjusted R^2^ of the model was 0.8637, while the predicted R^2^ was 0.6983; this difference may be attributed to minor experimental fluctuations and random errors in individual experimental runs, which is reasonable in the complex mechanical shearing grinding separation system. Notably, the Adeq Precision of the model reached 12.729 (far greater than the critical value of 4), and the CV% was 2.26 (less than 10), which collectively indicated high reliability and an adequate signal for reliable prediction.

**TABLE 5:** Variance analysis of test results.

Source	Sum of squares	df	Mean square	*F*	*P*
Model	606.13	14	43.30	13.67	<0.0001***
*X*_1_	127.60	1	127.60	40.30	<0.0001***
*X*_2_	91.69	1	91.69	28.96	<0.0001***
*X*_3_	56.38	1	56.38	17.80	0.0009***
*X*_4_	101.68	1	101.68	32.11	<0.0001***
*X*_1_ *X*_2_	14.29	1	14.29	4.51	0.0519*
*X*_1_ *X*_3_	0.0702	1	0.0702	0.0222	0.8837
*X*_1_ *X*_4_	11.56	1	11.56	3.65	0.0767*
*X*_2_ *X*_3_	11.63	1	11.63	3.67	0.0760*
*X*_2_ *X*_4_	0.0030	1	0.0030	0.0010	0.9758
*X*_3_ *X*_4_	0.1764	1	0.1764	0.0557	0.8168
$X_{1}^{2}$	20.91	1	20.91	6.60	0.0222**
$X_{2}^{2}$	92.58	1	92.58	29.24	<0.0001***
$X_{3}^{2}$	131.96	1	131.96	41.68	<0.0001***
$X_{4}^{2}$	12.50	1	12.50	3.95	0.0669*
Residual	44.33	14	3.17	​	​
Lack of fit	30.25	10	3.03	0.8596	0.6169
Pure error	14.08	4	3.52	​	​
Cor total	650.46	28	​	​	​

*P* < 0.01 (highly significant, ***); 0.01 < *P* < 0.05 (significant, **); 0.05 < *P* < 0.1 (slightly significant, *).

The influence of *X*_1_, *X*_*2*_, *X*_3_, *X*_4_, *X*_22_ and *X*_32_ on the separation rate *Y* of eggshell membrane in the model was highly significant; The effect of *X*_12_ on the separation rate *Y* of eggshell membrane was significant; The effect of *X*_1_ *X*_2_, *X*_1_ *X*_4_, *X*_2_ *X*_3_ and *X*_42_ on the separation rate *Y* of eggshell membrane was slightly significant; Other items were not significant. After removing insignificant terms, the regression equation for the influence of various factors on the separation rate of eggshell membrane was obtained , as shown in Equation 11:

$$Y=83.32+3.26X_{1}+2.76X_{2}+{2.17X}_{3}‐2.91X_{4}+1.89X_{1}X_{2}$$

$$‐1.70X_{1}X_{4}+1.71X_{2}X_{3}‐1.80X_{1}^{2}‐3.78X_{2}^{2}‐4.51X_{3}^{2}‐1.39X_{4}^{2}$$ (11)

where *Y* is the predicted eggshell membrane separation rate (%); *X*_1_ is the coded level of water content (%, −1 = 5, 0 = 10, 1 = 15); *X*_2_ is the coded level of motor rotational speed (r/min, −1 = 750, 0 = 900, 1 = 1,050); *X*_3_ is the coded level of feeding amount (g, −1 = 45, 0 = 50, 1 = 55); *X*_4_ is the coded level of gap between rotor and stator (mm, −1 = 1, 0 = 2, 1 = 3); *X*_1_ *X*_2_, *X*_1_ *X*_4_, *X*_2_ *X*_3_ are the interactive terms of experimental factors; $X_{1}^{2}$ , $X_{2}^{2}$ , $X_{3}^{2}$ , $X_{4}^{2}$ are the quadratic terms of experimental factors.

### 3.3 Mutual interactions between the significant factors

In order to visually and statistically analyze the relationship between the separation rate of eggshell membrane and various factors, the response surface and contour plots of significant interaction terms (*P* < 0.1) were obtained using Design-Expert 13 software, with the statistical results derived from the ANOVA in Table 5.

#### 3.3.1 Interaction of water content and motor rotational speed

The interaction term *X*_1_ *X*_2_ was slightly significant (*P* = 0.0519, correlation coefficient *r* = 0.38), indicating that the combined effect of water content and motor rotational speed on the separation rate cannot be ignored. Figure 11 shows the response surface and contour plot of their interaction under the condition that other factors are at the 0 level.

**FIGURE 11:**
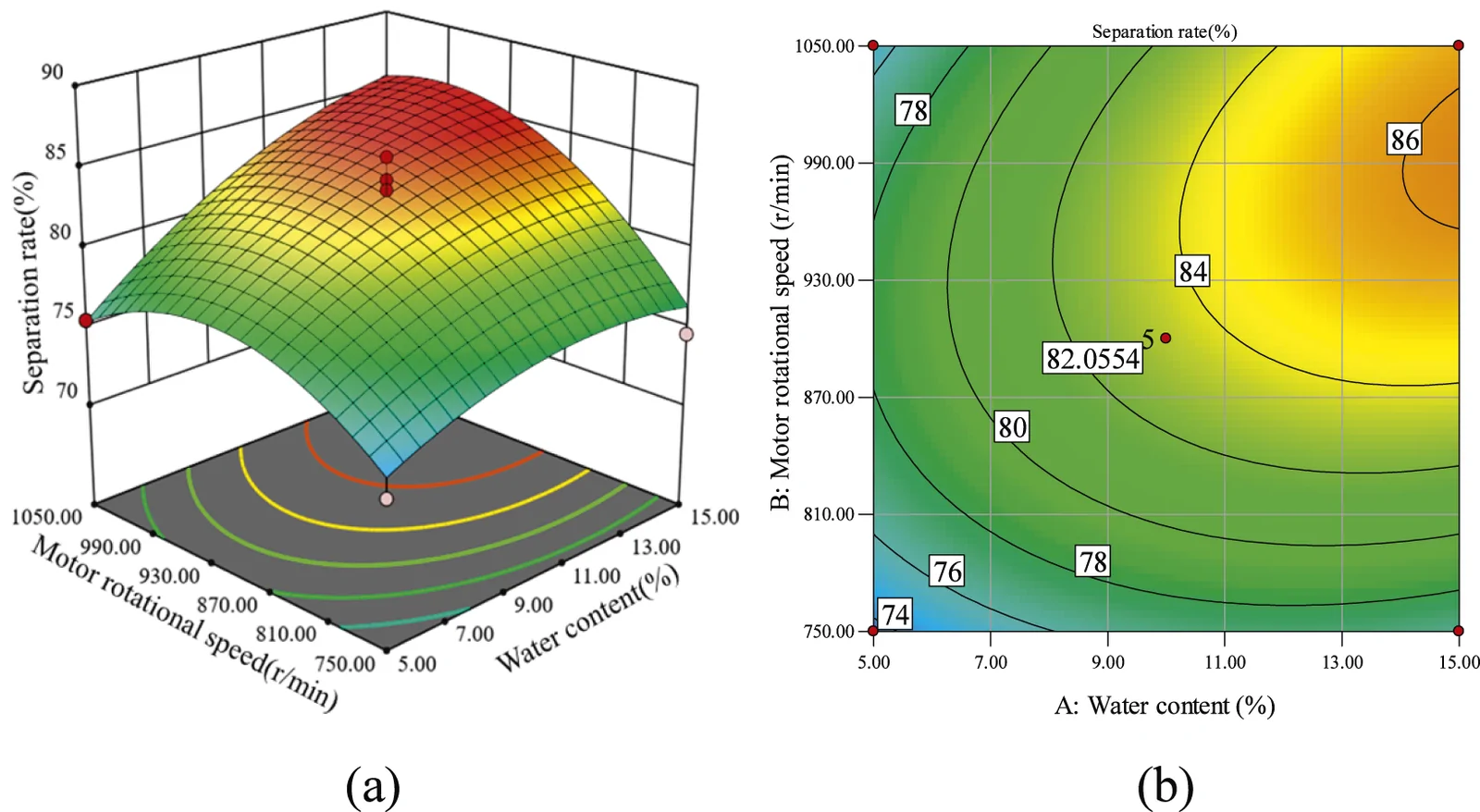
Response surface plot and contour plot of the interaction between water content and motor rotational speed. Note: P = 0.0519, r = 0.38. **(a)** Response surface plot. **(b)** Contour plot.

As illustrated in the response surface plot, the separation rate increases significantly with rising water content at low water content levels. This is because higher water content can improve the toughness of the eggshell membrane, which facilitates the separation of eggshell and eggshell membrane and prevents the eggshell membrane from breaking into small fragments, thereby enhancing collection efficiency. However, when water content exceeds a certain threshold, the separation rate still increases but the amplitude is relatively small, as the eggshell membrane’s moisture saturation limits further improvement.

At a fixed water content, the separation rate first rises and then declines with increasing motor rotational speed. At low speeds, the motor cannot fully overcome the binding force between the eggshell and eggshell membrane, reducing separation efficiency. Conversely, excessive speed causes excessive fragmentation of the eggshell and eggshell membrane, complicating later collection. The contour plot exhibits an elliptical shape, which further confirms the significant interaction between the two factors.

#### 3.3.2 Interaction of water content and gap between rotor and stator

The interaction term *X*_1_ *X*_4_ was slightly significant (*P* = 0.0767, correlation coefficient *r* = 0.35), suggesting a notable combined effect of water content and rotor-stator gap on the separation rate. Figure 12 presents the corresponding response surface and contour plot.

**FIGURE 12:**
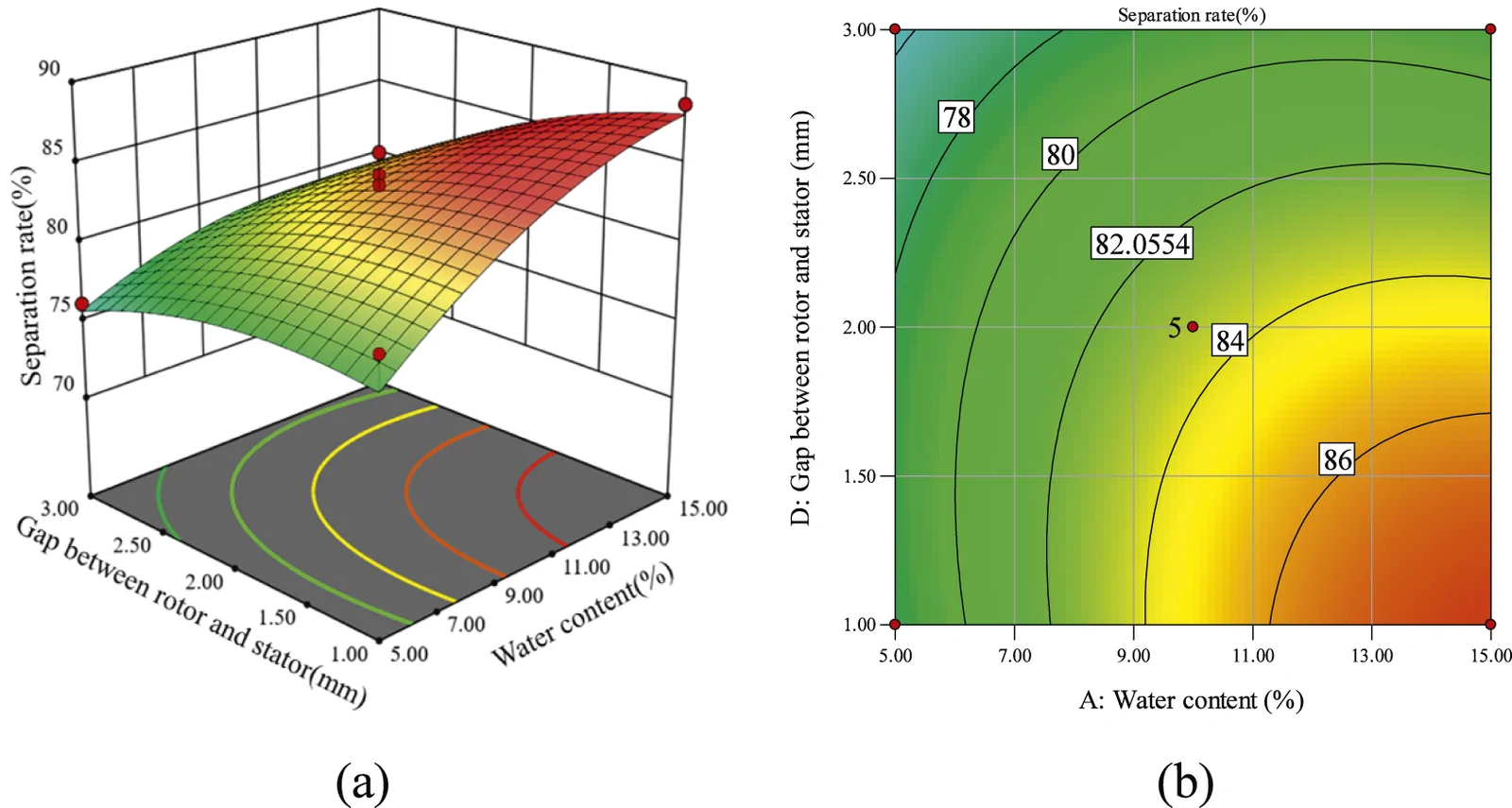
Response surface plot and contour plot of the interaction between water content and gap between rotor and stator. Note: P = 0.0767, r = 0.35. **(a)** Response surface plot. **(b)** Contour plot.

It can be seen from the response surface that when the water content is constant, the separation rate decreases with the increase of the gap; when the gap is constant, the separation rate increases with the increase of water content. When the gap is greater than the 0 level (2 mm), the separation rate decreases significantly with the increase of the gap. This is because when the gap is too large, the discarded eggshell cannot undergo sufficient shearing and grinding action, resulting in a rapid decrease in separation rate. The contour plot shows a moderate elliptical trend, consistent with the statistical result of slight significance for the interaction term.

#### 3.3.3 Interaction of motor rotational speed and feeding amount

The interaction term *X*_2_ *X*_3_ was slightly significant (*P* = 0.0760, correlation coefficient *r* = 0.35), indicating that the interaction between motor rotational speed and feeding amount has a non-negligible impact on the separation rate. Figure 13 displays the response surface and contour plot of their interaction.

**FIGURE 13:**
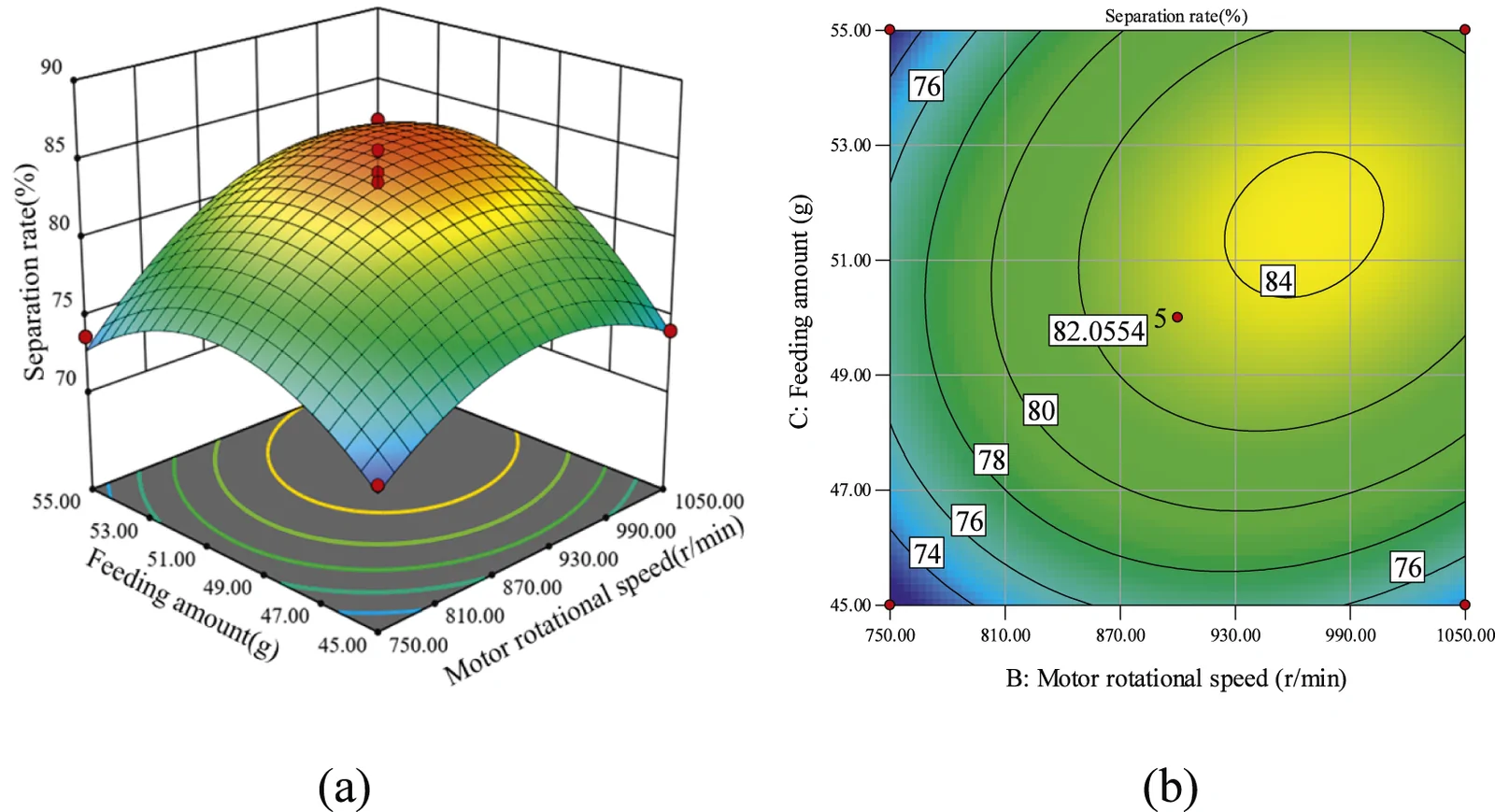
Response surface plot and contour plot of the interaction between motor rotational speed and feeding amount. Note: P = 0.0760, r = 0.35. **(a)** Response surface plot. **(b)** Contour plot.

As shown in the response surface, when the feeding amount remains constant, the separation rate first increases and then decreases with the increase of rotational speed; when the speed remains constant, the separation rate first increases and then decreases with the increase of feeding amount. When both factors are at the intermediate level (rotational speed = 900 r/min, feeding amount = 50 g), the overall separation rate is the highest. This is because the device designed in this study is a laboratory-scale device with limited internal space. When the feeding amount is too high, part of the discarded eggshell cannot undergo effective crushing, which affects the separation rate. The elliptical contour plot further verifies the slight significance of the interaction between the two factors.

### 3.4 Parameter optimization and validation

To achieve the highest separation rate of eggshell membrane and obtain the optimal combination of experimental factors, the Design-Expert 13 software was used to optimize the experimental parameters. The optimization conditions obtained from the response surface analysis results are shown in Equation 12:

$$\left\{\begin{array}{c} \max Y\left(X_{1},X_{2},X_{3},X_{4}\right)\\ 10\%{\leq X}_{1}\leq 15\%\\ 750\;r/\min{\leq X}_{2}\leq 1050\;r/\min\\ 45\;g\leq X_{3}\leq 55\;g\\ 1\text{ mm}{\leq X}_{4}\leq 2\text{ mm} \end{array}\right.$$ (12)

The optimal parameter combination of each factor was determined through the above optimization. When the water content was 15%, the motor rotational speed was 991 r/min, the feeding amount was 51.05 g, and the gap between rotor and stator was 1.48 mm, the eggshell membrane separation rate reached the highest of 88.72%. Considering the operability of the experiment, the optimal feeding amount calculated by the model (51.05 g) was rounded to 51 g, and the motor rotational speed (991 r/min) and gap between rotor and stator (1.48 mm) were adjusted to 1,000 r/min and 1 mm respectively for practical operation. Therefore, the water content of 15%, motor rotational speed of 1,000 r/min, feeding amount of 51 g, and gap between rotor and stator of 1 mm were selected as the experimental conditions for three repeated validation tests.

Three replicate measurements were conducted, and the eggshell membrane separation rates were 86.97%, 87.25%, and 87.62%, with an average of 87.28%. The average value is close to the predicted optimization result, indicating that the optimization result is reliable.

## 4 Discussion

A shearing grinding integrated separation device was specifically designed to take advantage of the distinct mechanical properties of eggshell (high brittleness) and eggshell membrane (high toughness), addressing the drawbacks of traditional separation methods. This section discusses the study’s findings by comparing with previous research, elaborating on the separation mechanism, and analyzing limitations and future directions.

### 4.1 Comparison with previous separation methods

Unlike conventional impact or stirring-based mechanical separation devices reported in previous studies, the custom-designed shearing-grinding unit in this work integrates shear and grinding effects. Its tailored rotor-stator structure makes full use of the mechanical differences between brittle eggshell and tough eggshell membrane, achieving targeted fragmentation rather than random impact crushing. This dedicated structural design constitutes the main novelty of the proposed device.

The eggshell membrane separation rate of 87.28% achieved in this study is competitive with existing technologies. Compared to physical methods, the shearing grinding method outperforms mechanical stirring and flash evaporation—with no risk of protein denaturation and simpler equipment structure. While chemical methods can reach ∼90% separation rate, they cause severe environmental pollution and waste eggshell resources, whereas our method is green and recovers both eggshell and membrane without modification. Enzymatic hydrolysis requires high-cost reagents and strict process control, which our method avoids by using only mechanical forces, reducing operational complexity and costs. Nevertheless, this mechanical shearing-grinding method also has limitations. Its separation performance is susceptible to raw material moisture, and further work is required to improve the separation rate to match high-efficiency chemical routes.

### 4.2 Mechanistic explanation of the shearing grinding separation process

The superior separation performance stems from the synergistic effect of shearing and grinding, leveraging the mechanical property differences between eggshell and membrane. First, the 50° shearing blades generate targeted shearing forces to break the glycoprotein-based adhesive layer between eggshell and membrane, weakening their binding. Second, the grinding blades and rolling ring exert grinding forces that selectively crush brittle eggshell into small particles, while the tough eggshell membrane remains intact due to its high elasticity. This “shearing to disrupt adhesion + grinding to crush eggshell” synergism ensures efficient and selective separation—without shearing, the adhesive layer remains intact; without grinding, eggshell fragments adhere to the membrane, reducing separation efficiency.

The separation mechanism relies on the distinct mechanical behaviors of eggshell and eggshell membrane. Eggshell exhibits high brittleness and low fracture toughness, which makes it easily fragmented under combined shear and grinding loads. In contrast, eggshell membrane possesses good tensile toughness and ductility, enabling it to resist crushing and maintain its integrity under the same loading conditions (Ahmed et al., 2021). The designed rotor–stator structure produces targeted shear and abrasive forces, breaking brittle eggshell fragments while protecting the tough membrane from over-destruction, thereby realizing effective physical separation.

### 4.3 Limitations and future research directions

This study has limitations: it is laboratory-scaled and only tested on fresh Hy-Line Brown eggshells. Future research will focus on three aspects: (1) Scaling up the device to improve batch processing capacity; (2) Adapting the method to eggshells of different species and storage conditions (e.g., dried, refrigerated) by adjusting operational parameters; (3) Optimizing key components with wear-resistant composites to enhance long-term stability. Additionally, numerical simulation could be used to further refine blade angle and grinding gap for industrial application. Moreover, further characterization including morphology, purity and compositional detection of separated eggshell and eggshell membrane was not carried out in the current work. Supplementary characterization tests will be performed in future studies to further evaluate the quality of separated products. Systematic evaluation of energy consumption, operating cost and economic feasibility for industrial scale-up will be carried out in future scaled-up trials.

To further intuitively compare the characteristics of the proposed shearing-grinding strategy with commonly reported eggshell and eggshell membrane separation approaches, a comparative summary is presented in Table 6.

**TABLE 6:** Comprehensive comparison of different eggshell and eggshell membrane separation technologies.

Separation method	Separation principle	Advantages	Limitations	Separation performance
Mechanical stirring	Realize separation through random impact and stirring force	Simple operation, no chemical pollution	Single force form, easy to cause membrane damage, low separation targeting	Moderate separation efficiency, poor stability
Flash evaporation	Separation by instantaneous steam impact	Clean process, no chemical additives	High energy consumption, strict equipment requirements	Separation rate around 73.48%
Acid/alkaline immersion (chemical)	Dissolve interfacial binding substances using chemical reagents	High separation rate	Acid-base wastewater pollution, waste of eggshell resources	Maximum separation rate of approximately 90%
Enzymatic hydrolysis (biological)	Degrade interfacial components via specific enzymes	Mild reaction conditions, high product quality	Expensive enzyme reagents, long reaction time, strict reaction control	Separation rate around 88%
This study (shearing-grinding method)	Targeted separation relying on mechanical property differences: Brittle eggshell breaks under combined shear and grinding, while tough eggshell membrane remains intact	Green, low cost, simple device structure, both eggshell and membrane can be recycled	Separation effect is sensitive to raw material moisture content, requiring further optimization	Maximum separation rate of 87.28%

## 5 Conclusion

This study developed a shearing grinding device for eggshell and eggshell membrane separation, leveraging their distinct mechanical properties. Based on single-factor experiments and response surface optimization, the optimal parameters were determined to be: 15% water content, 1,000 r/min motor speed, 51 g feeding amount, and 1 mm rotor-stator gap, achieving a maximum separation rate of 87.28%. The device’s key components work synergistically to disrupt the adhesive layer and selectively crush eggshell, enhancing separation efficiency. This approach is environmentally friendly, preserves material intrinsic properties, and features simple equipment with low energy consumption. The findings provide a practical green reference for industrial utilization of eggshell waste, with future potential in device scaling and broader application.

## Data Availability

The original contributions presented in the study are included in the article/Supplementary Material, further inquiries can be directed to the corresponding author.
