# Supplementary material for: Design and optimization of a shearing grinding device for efficient separation of eggshell and eggshell membrane
Source: Front Bioeng Biotechnol. 2026 Sep 21;14:1928807. doi: 10.3389/fbioe.2026.1928807 (PMC13634946; doi:10.3389/fbioe.2026.1928807)
Supplement: Supplementary file 1 [file DataSheet1.docx]

| Std | Egg  membrane  weight(g) | **Water content**  ***X*_1_ (%)** | **Motor rotational speed**  ***X*_2_ (r/min)** | **Feeding amount**  ***X*_3_ (g)** | **Gap between rotor and stator**  ***X*_4_ (mm)** | **Separation rate**  ***Y*(%)** |
| --- | --- | --- | --- | --- | --- | --- |
| 1 | 1.1013 | 5 | 750 | 50 | 2 | 72.38 |
| 2 | 1.1347 | 15 | 750 | 50 | 2 | 74.58 |
| 3 | 1.1481 | 5 | 1050 | 50 | 2 | 75.46 |
| 4 | 1.2966 | 15 | 1050 | 50 | 2 | 85.22 |
| 5 | 1.0393 | 10 | 900 | 45 | 1 | 75.90 |
| 6 | 1.3528 | 10 | 900 | 55 | 1 | 80.83 |
| 7 | 0.9848 | 10 | 900 | 45 | 3 | 71.92 |
| 8 | 1.3003 | 10 | 900 | 55 | 3 | 77.69 |
| 9 | 1.2212 | 5 | 900 | 50 | 1 | 80.26 |
| 10 | 1.3484 | 15 | 900 | 50 | 1 | 88.62 |
| 11 | 1.1582 | 5 | 900 | 50 | 3 | 76.12 |
| 12 | 1.1819 | 15 | 900 | 50 | 3 | 77.68 |
| 13 | 0.9902 | 10 | 750 | 45 | 2 | 72.31 |
| 14 | 1.0141 | 10 | 1050 | 45 | 2 | 74.06 |
| 15 | 1.2328 | 10 | 750 | 55 | 2 | 73.66 |
| 16 | 1.3762 | 10 | 1050 | 55 | 2 | 82.23 |
| 17 | 0.9817 | 5 | 900 | 45 | 2 | 71.69 |
| 18 | 1.0962 | 15 | 900 | 45 | 2 | 80.05 |
| 19 | 1.2439 | 5 | 900 | 55 | 2 | 74.32 |
| 20 | 1.3926 | 15 | 900 | 55 | 2 | 83.21 |
| 21 | 1.2070 | 10 | 750 | 50 | 1 | 79.33 |
| 22 | 1.2773 | 10 | 1050 | 50 | 1 | 83.95 |
| 23 | 1.1110 | 10 | 750 | 50 | 3 | 73.02 |
| 24 | 1.176 | 10 | 1050 | 50 | 3 | 77.53 |
| 25 | 1.2300 | 10 | 900 | 50 | 2 | 80.84 |
| 26 | 1.2501 | 10 | 900 | 50 | 2 | 82.16 |
| 27 | 1.2822 | 10 | 900 | 50 | 2 | 84.27 |
| 28 | 1.2727 | 10 | 900 | 50 | 2 | 83.65 |
| 29 | 1.3036 | 10 | 900 | 50 | 2 | 85.68 |
